# Altering Dietary Soluble Protein Levels With Decreasing Crude Protein May Be a Potential Strategy to Improve Nitrogen Efficiency in *Hu* Sheep Based on Rumen Microbiome and Metabolomics

**DOI:** 10.3389/fnut.2021.815358

**Published:** 2022-01-18

**Authors:** Zhenbin Zhang, Khuram Shahzad, Sijun Shen, Rong Dai, Yue Lu, Zhiqi Lu, Chuang Li, Yifei Chen, Ruxin Qi, Pengfei Gao, Qingyong Yang, Mengzhi Wang

**Affiliations:** ^1^College of Animal Science and Technology, Yangzhou University, Yangzhou, China; ^2^State Key Laboratory of Sheep Genetic Improvement and Healthy Production, Xinjiang Academy of Agricultural Reclamation Sciences, Shihezi, China; ^3^Department of Biosciences, COMSATS University Islamabad, Islamabad, Pakistan; ^4^College of Animal Science and Technology, Shihezi University, Shihezi, China

**Keywords:** soluble protein, low-protein diet, nitrogen metabolism, rumen microbiome, metabolomics, *Hu* sheep

## Abstract

Ruminants account for a relatively large share of global nitrogen (N) emissions. It has been reported that nutrition control and precise feeding can improve the N efficiency of ruminants. The objective of the study was to determine the effects of soluble protein (SP) levels in low-protein diets on growth performance, nutrient digestibility, rumen microbiota, and metabolites, as well as their associations of N metabolism in fattening *Hu* sheep. Approximately 6-month-old, 32 healthy fattening male *Hu* sheep with similar genetic merit and an initial body weight of 40.37 ± 1.18 kg were selected, and divided into four groups (*n* = 8) using the following completely randomized design: the control diet (CON) with a 16.7% crude protein (CP) content was prepared to meet the nutritional requirements of fattening sheep [body weight (BW): 40 kg, average daily gain (ADG): 200–250 g/d] according to the NRC recommendations; other three include low protein diets (LPA, LPB, and LPC) of CP decreased by ~10%, with SP proportion (%CP) of 21.2, 25.9, and 29.4 respectively. The feeding trial lasted for 5 weeks including the first week of adaptation. The results showed no difference in the growth performance (*P* > 0.05); DM and CP digestibility were higher in LPB and LPC, with maximum organic matter digestibility in LPB (*P* < 0.05). Low-protein diets decreased serum urea-N whereas urinary urea-N was lower in LPB and LPC (*P* < 0.05), while N retention and the biological value of N were higher in LPB and LPC (*P* < 0.05). Ruminal NH_3_-N concentration in LPA and LPB was low than CON (*P* < 0.05), while total volatile fatty acid (TVFA), acetate, propionate, and butanoate were all lowest in LPA (*P* < 0.05). In the rumen microbiome, LPB increased the community richness in Prevotellaceae and *Prevotella_1* (*P* < 0.05); Metabolomics analysis revealed low-protein diets downregulated the amino acid metabolism pathways, while the biosynthesis of unsaturated fatty acids along with vitamin B6 metabolism were upregulated with increased SP. These findings could help us understand the role of different SP levels in the regulation of rumen microbial metabolism and N efficiency. Overall, low-protein diets (CP decreased by ~10%) can reduce serum urea-N and ruminal NH_3_-N without affecting the growth performance of fattening *Hu* sheep. Additionally higher N efficiency was obtained with an SP proportion of ~25–30%.

## Introduction

Animal production systems (specifically, ruminants) produce a large proportion of ammonia (NH_3_) and nitrous oxide (N_2_O) into the atmosphere, where >70% of feed nitrogen (N) is released (1, 2). The sources of gaseous N emissions include urine and feces discharged by animals during grazing or captivity (3). It has been anticipated that the N_2_O emission from animals and their wastes is 2.7 Tg (Tg = 10^12^g) per annum, which is around 30–50% of total agricultural N_2_O emissions. Similarly, NH_3_ release is about 22−32 Tg, accounting for 50–75% of the total NH_3_ emissions, affecting overall human health and environmental issues (4, 5).

In livestock practices, a number of researchers have demonstrated that reducing dietary crude protein (CP) content is an important way to minimize N emissions and alleviate protein feed resources [e.g., pig (6), poultry (7), and ruminants (8)]. To our knowledge, under normal dietary conditions, the N utilization of ruminants is lower than that of monogastric animals, where only about 20–40% N is retained (9). The NRC (10) classifies the optimal utilization of dietary CP which requires the selection of suitable feed protein sources to provide the type and quantity of rumen degradable protein (RDP) that meets but does not exceed the rumen microbial N requirements. Arguably, the amount and degradability of dietary protein are the keys to affect rumen fermentation and nutrient absorption (11).

Soluble protein (SP) is defined as the protein fraction A dissolved in borate-phosphate buffer (12). It is composed of ammonia, nitrate, amino acids, peptides, and some true proteins, which can provide rumen microorganisms with rapid degradable protein and N (within 1 h after intake) (13). On the other hand, insoluble protein (ISP) is the sum of protein fractions B and C, including potential RDP and rumen undegradable protein (UDP) (12). Potential RDP cannot be utilized immediately like SP, but it will slowly degrade with time. Due to lignin-bound proteins, pass rates, and feeding management, not all potential RDP may be degraded in the rumen (14). Obviously, the utilization of protein and other nutrients is affected by the type and quantity of each protein fraction. Moreover, adjusting dietary SP proportion has the potential to improve N utilization in ruminants (15). In previous studies, dietary SP (%CP) increased from 34.4 to 44.9 in dairy cattle, which increased the milk urea N and plasma urea N but decreased the CP and true protein in milk (16). Meanwhile, Majdoub et al. (17) found that by increasing dietary SP (%CP) from 22 to 44, the dry matter intake (DMI) of dairy cattle was not affected, but milk yield and N utilization were decreased. Davis (18) reported that feeding a diet containing CP of 15.5–16.5% and SP (%CP) of 30–35% (without urea) to dairy cattle, can increase the milk yield.

The ruminant foregut fermenter is considered to be an important microbial ecosystem, where the rumen microbiome plays an important role in balancing between food safety and environmental impact (19). Diet influenced rumen microbes and their metabolites significantly, upregulation of the fiber-degrading microorganisms improved the production of volatile fatty acid (VFA) that can be absorbed by the host (20). Different diets can affect the harmful intermediate accumulation of fermentation in the rumen, such as lactate, which was produced under high-concentration feeding. The capability of ruminants to effectively utilize dietary nutrients is closely related to the dietary ingredients, and to the composition and structure of rumen microbes. Therefore, clarifying the role of the rumen microbiome in shaping the physiological parameters of the host can increase the agricultural yield and reduce environmental pollution by adjusting the structure of bacterial communities (21).

Even though reducing dietary CP levels has been widely required in livestock production to reduce N emissions and the feeding strategy of SP has also been investigated on sustainable cattle production, however, to date, there are few reports on the effects of SP level on rumen microbes and N metabolism in ruminants, especially in low-protein diets. Furthermore, the interaction between diet–microbiome is yet to be revealed when sheep were fed by different SP levels. Herein, we hypothesized that altering SP levels in low-protein diets impact nutrient absorption and N excretion by modifying the rumen microbiota and metabolites in sheep. Therefore, the effects of SP levels in low-protein diets on rumen microbiota, metabolites, and N excretion of fattening *Hu* sheep were investigated in the present study, aiming to provide a potential approach to convert dietary N into sheep products more effectively and reasonably and limit N emissions.

## Materials and Methods

### Feed Preparation and the Determination of SP Fractions

Six feed samples were selected for the determination of SP fractions, including one roughage (mixed silage) and five concentrates (corn, soybean meal, wheat bran, urea, and corn protein meal) which were used in the feeding trial. Mixed silage consisted of cabbage and straw in the ratio of 6:4, collected from the experimental farm of Yangzhou University (Jiangsu, China); then these were cut into 2–3 cm in length, mixed with 0.035 g/kg *Lactobacillus plantarum* and 0.250 g/kg *cellulase*, and then sampled after 45 days of anaerobic fermentation. Concentrates were bought from commercial feed suppliers and sampled, respectively. Mixed silage samples were freeze-dried, whereas other feed samples were dried at 65°C in a forced-air oven for 24 h. All feeds were ground in a Retsch ZM 100 Wiley mill (Retsch GmbH, Haan, Germany) to pass through a 1 mm screen.

Soluble protein content was determined using the standardization of procedures for N fractionation (buffer-soluble N) described by Licitra et al. (22), which was recommended by the Cornell net carbohydrate-protein system (CNCPS). Concretely, weighed about 0.5 g ground dry sample into a 150 ml fermentation glass bottle from six samples respectively (3 replicates per feed sample), then 50 ml borate-phosphate buffer (pH 6.7–6.8, including monosodium phosphate, sodium tetraborate, and tertiary butyl alcohol) and 1 ml 10% sodium azide solution were added. Then the mixture was incubated in a thermostatic water bath shaker for 1 h at 39°C (SHA-A, Hengfeng Instrument, Jintan, China) with a shaking frequency of 120 rpm, and followed by filtration step through a filter paper (Whatman no. 54, UK). Afterward, the washing step with 250 ml of cold distilled water was performed to determine residual N by Kjeldahl to get the ISP fraction. Then, the SP contents were calculated by total CP min ISP calculation formulae. The protein fractions of the feed samples are presented in the Supplementary Table 1.

### Animals, Diet Treatment, and Experimental Design

Approximately 6-month-old, 32 healthy fattening male *Hu* sheep with similar genetic merit and an initial body weight (40.37 ± 1.18 kg) were selected; then, they were randomly divided into four groups (*n* = 8 each treatment) in a completely randomized design: CON was Control diet, CP was 16.7% based on the nutritional requirements of the fattening sheep [body weight (BW): 40 kg, average daily gain (ADG): 200–250 g/d] according to the NRC recommendations for sheep (23); LPA, LPB, and LPC were all low-protein diets with CP reduced by ~10%, where SP proportion (% of CP) was configured as 21.2 25.9, and 29.4%, respectively. Except for CON, the other three diets met 90% of the CP requirement, and the targeted SP proportion was mainly achieved through a different additional proportion of concentrate. The actual protein solubilities of the four complete diets were determined by the method explained in Section Feed Preparation and the Determination of SP Fractions. The ingredients and nutrition contents of the diets are shown in Table 1.

**Table 1:** Ingredients and nutritive levels of the experimental diets.

**Items**	**Treatments**			
	**CON**	**LPA**	**LPB**	**LPC**
**Ingredient, % of DM**				
Mixed silage^1^	50	50	50	50
Corn	34	35.5	35.65	38
Soybean meal	5	–	1.5	-
Wheat bran	8.5	11	11	10
Urea	–	–	0.2	0.5
Corn protein meal	1	2	0.15	–
NaHCO_3_	0.5	0.5	0.5	0.5
Premix^2^	0.5	0.5	0.5	0.5
NaCl	0.5	0.5	0.5	0.5
Total	100	100	100	100
**Nutritive level, g/kg**				
DE (MJ/ kg)^3^	14.73	14.65	14.81	14.76
CP	166.9	155.1	155.7	156.5
SP (% of CP)	23.5	21.2	25.9	29.4
EE	33.5	34.6	34.5	34.8
NDF	459.9	442.1	471.2	478.7
ADF	271.7	266.7	274.9	276.2
Ash	123.3	123.2	119.6	115.6
Ca	4.6	4.4	4.5	4.5
*P*	3.9	4.2	4.2	4.4

1 *Cabbage and straw were mixed in 6:4 silage ratio with the addition of 0.035 g/kg Lactobacillus plantarum and 0.250 g/kg cellulase*.

2 *Formulated to provide [per kg of dry matter (DM)] Vitamin A 130 KIU, Vitamin D3 30 KIU, Vitamin E 13 KIU, Fe 0.8 g, Mn 1.0 g, Zn 3.0 g, Cu 0.2 g, Se 8 mg, and NaCl 100 g*.

3 *Calculated according to nutrient requirements of fattening sheep*.

*DE, digestible energy; CP, crude protein; SP, soluble protein; EE, ether extract; NDF, neutral detergent fiber; ADF, acid detergent fiber; Ca, calcium; P, phosphorus*.

*Treatments: CON is 16.7% CP based on nutritional requirements, CP of LPA, LPB, and LPC is decreased by ~10%, SP proportion (% of CP) 21.2, 25.9, and 29.4, respectively*.

Thirty-two sheep were individually housed in a covered pen, and all the diets were fed as total mixed ration (TMR) with a forage-to-concentrate ratio of 50:50 (DM basis) twice daily in equal amounts (08:00 and 18:00 h). The amount of DM of the feed offered to the sheep was about 3% of the BW. The feeding trial lasted for 5 weeks, consisting of 1 week of adaption and 4 weeks of experimental periods. The animals were given fresh drinking water throughout the whole experimental period. Before starting the experiment, the sheep were vaccinated to avoid infectious diseases and then dewormed.

### Sample Collection and Measurement

#### Growth Performance Indicators

The quantities about the feeds offered and refused by each individual *Hu* sheep were measured before the morning feed throughout the whole feeding trial to calculate DM intake (DMI). The body weights of all sheep were calculated weekly during the early morning before feeding. Here ADG was the regression of BW measured with the passage of time. The feed requirement of each sheep was measured using feed conversion rate (FCR), which is the ratio of DMI to ADG.

#### Feces and Urine Sampling and Analysis

To measure the apparent total tract digestibility (ATTD), feed samples and refused samples were gathered once every week. Then feces of each sheep were collected in fecal bags at the mid-term (from day 12 to 14) and the end-term (from day 26 to 28) of the feeding trial and weighted score. At each sampling date of two periods, around 10% of feces samples of each sheep were collected by adding a solution of H_2_SO_4_ (10% vol/vol) to prevent N loss during storage. After drying to a constant mass at 65°C in a forced-air oven (DHG9626A, Jinhong Co., Ltd, Shanghai, China), the feed, refusal, and fecal samples were ground in such a way that these can pass through a 1 mm screen with a Retsch ZM 100 Wiley mill (Retsch GmbH, Haan, Germany). By following the Association of Official Analytical Chemists (24), the chemical analysis for DM, (ID 973.18), crude ash (ID 923.03), and crude protein (CP, ID 4.2.08) was performed. Other contents, such as neutral detergent fiber (NDF) and acid detergent fiber (ADF) were determined by a filter bag technique (ANKOM 2000; Ankom Technology Corp., Fairport, NY) as explained by Van Soest et al. (25). The percent of ATTD was calculated by using the following formula:


$$\text{ATTD}\left(\%\right)=\left(\text{1}-\frac{\text{FNR}}{\text{FNI}}\right)\times 100\%$$

Where FNR is fecal nutrition retained (g DM/d) and FNI is feed nutrient intake (g DM/d).

The urine of each sheep was collected at the same time as the feces were collected in the middle and at the end of the experimental periods to determine the N balance. Total urine was collected once every day using a handmade urine bag into a container pre-filled with H_2_SO_4_ (10% vol/vol) to reduce ammonia loss. Acidified urine pH was checked with a portable pH instrument (PB-21, Sartorius Scientific Instrument Co., Ltd. Goettingen, Germany) to keep the pH below 3.0 (26). Approximately, 10% of the daily urine was stored at −20°C for chemical analysis. The N content of urine was determined using a Kjeldahl analyzer (Kjeltec 2300; FOSS Analytical AB, Hoganas, Sweden), and the N content of dietary and fecal material were calculated using the 6.25 factor with CP, using the following formula:


CP(g/kg)=N(g/kg)× 6.25

Urinary urea N was analyzed using a urea assay kit (C013-2, Jiancheng Bioengineering Institute, Nanjing, China) according to the study by Ding et al. (27). The biological value of N (BVN) was calculated using the following formula:


$$\text{BVN }(\%)=\frac{\text{NR}}{\text{NI}-\text{FNE}}\times 100\%$$

Where NR is N retention (g DM/d), NI is N intake (g DM/d), and FNE is Fecal N Excretion (g DM/d).

#### Blood Sampling and Analysis

During the last day of the trial, the blood samples of each sheep were collected from jugular veins, using the venipuncture method, into tubes containing a coagulant, before the morning feed. The serum was separated after centrifugation (3,000 g, 15 min, 4°C) and then reserved at −20°C in a freezer for later analysis purposes. The serum urea N was analyzed according to the method of urinary urea N.

### Rumen Fluid Sampling and Analysis

Two hours after feeding of the last day in the trial, rumen fluid samples (30 mL) were taken from randomly selected six sheep of each group by using an oral stomach tube having a length of 1.2 m and a width of 1.0 cm. This tube was connected to an adjustable vacuum pump. Around 20 ml of rumen sample containing saliva contamination was discarded and the remaining fluid was filtered through four layers of cheesecloth followed by immediate readings of pH values. Later, these samples were stored at −80°C for further analysis of different compounds, such as ammonia N (NH_3_-N), microbial crude protein (MCP), VFA, microbial DNA extraction, and metabolomic analysis.

The concentration of NH_3_-N was measured using phenol-sodium hypochlorite colorimetry according to Weatherburn (28); MCP was determined using the trichloroacetic acid precipitation method described by Zhang et al. (29); VFA concentrations were determined by gas chromatography (GC-14B, Kyoto, Japan) according to the study by Wang et al. (30). The model of the GC capillary column (CP-Wax 52 CB, Agilent, US) was 30 m (length) × 0.53 m (diameter) × 1 μm (film thickness), the temperature of the injector and detector was set at 200°C; the initial temperature of the capillary column was 100°C, later the temperature was increased to 150°C at 3°C/min, the sensitivity was set to 10^1^, and the attenuation was set to 2^5^.

### DNA Extraction From Rumen Fluid Samples, PCR Amplification, 16S rRNA Sequencing, and Data Mining

Genomic DNA was extracted from rumen fluid samples by using HiPure Soil DNA Kit (D3142B, Magen Biotechnology Co., Ltd., Guangzhou, China). Nanodrop 2000 Spectrophotometer (Thermo Fisher Scientific, Wilmington, US) was used to determine the concentration and purity of genomic DNA. The gene amplification of the region V3–V4 of the 16S rRNA was performed by PCR using a set of primers, such as 341F (5′-CCTACGGGNGGCWGCAG-3′) and 806R (5′-GGACTACHVGGGTWTCTAAT-3′). The PCR amplification program was performed using GeneAmp® 9700 PCR instrument (ABI, CA, US) by following the factors, such as an initial denaturation at 95°C for 5 min; 27 cycles of 95°C for 30 s, annealing at 55°C for 30 s, elongation at 72°C for 45 s; and a final extension at 10 min at 72 and 10°C, until halted. PCR reactions were carried out in 2 × Phanta Master Mix, 30 μl reaction mixture, containing 15 ul 2 × Phanta Master Mix, 1 ul Bar-PCR primer F(10 uM), 1 ul Primer R (10 uM), 10–20 ng Genomic DNA and finally added with ddH_2_O to 30 μL. After PCR was completed, the amplified PCR products were extracted by 2% agarose gel (voltage: 80 v; electrophoresis time: 40 min) to determine the target band size and concentration.

The amplified fragments were then sent to Genepioneer Biotechnologies Co., Ltd (Nanjing, China) for 16S rRNA sequencing using Illumina Novaseq PE250 platform to obtain paired-end (PE) reads. Pandaseq software (31) was used to merge pairs of reads into a sequence according to the overlap relationship between PE reads, with a minimum overlap length of 10 bp. The reads with tail quality values below 20 were filtered out using PRINSEQ software (32). Those sequences whose N length accounts for 5% of the total length of the sequence were also filtered out. Chimera sequences were removed with Usearch (33) by the Denovo method. Uparse software was employed to cluster unique sequences with 0.03 cutoff (97% similarity) into representative operational taxonomic units (OTUs) (34). To classify all OTUs with a confidence threshold of 0.8, the Ribosome Database Program (RDP) was used. Later on, the Silva132 database (http://www.arb-silva.de/) was employed to classify them into the species level. Based on the abundance and annotation information of OTU, the sequence proportion of each sample was counted at each classification level (phylum, class, order, family, and genus) to the total sequence number, and finally the dominant flora of phylum, family, and genus level were selected to visualize through the ggplot2 package in R program (v 3.6.1).

Community richness (ACE and Chao1 index), community diversity (Shannon and Simpson index), and community coverage (Goods coverage) was calculated using the “vegan” R package; “UpSetR” package was used to show core OTUs, dispensable OTUs, and specific OTUs; Principal coordinates analysis (PCoA) was performed and treatment differences based on Bray–Curtis dissimilarity matrix were determined to reveal the differences in the bacterial communities across the four treatments and plotted using the “ggplot2” package. Analysis of similarities (ANOSIM) was employed to indicate group similarity and Adonis was employed to describe the strengths and significance of the differences among the microbial communities, where 0 represents indistinguishable and 1 represents dissimilar (35). For the two analyses, the *P*-values were determined based on 999 permutations. The linear discriminant analysis (LDA) effect size (LEfSe) was used to identify the most differentially abundant bacterial taxa among treatments (http://huttenhower.sph.harvard.edu/galaxy). Effect-size threshold of 2 and a significance level of *P* < 0.05 were applied to identify the biomarker taxa.

### Nontargeted Metabonomics Based on Liquid Chromatograph-Mass Spectrometer and Data Processing

The liquid chromatograph-mass spectrometer (LC-MS) platform was used to analyze 24 rumen samples by following the procedure as previously described by Ma et al. (36) with some modifications. All analyses were performed using an ACQUITY UPLC system (Waters Corporation, MD, USA) coupled with an Orbitrap Q Exactive HF-X mass spectrometer (Thermo Fisher Scientific, MA, USA). Briefly, 5 μL sample was taken and injected into the column (2.1^*^100 mm, 1.8 μm; Waters ACQUITY UPLC HSS T3, US) with chromatographic separation conditions, such as column temperature set to 40°C; mobile phase A with water, and 0.05% of formic acid; mobile phase B with acetonitrile; flow rate set to 0.3 mL/min; and mobile phase gradient time with 16 min. Finally, the Q Exactive HF-X mass spectrometer was operated using the positive/negative mode with a temp of 300°C, sheath gas flow rate of 45 arb, aux gas flow rate of 15 arb, sweep gas flow rate of 1arb, spray voltage of 3.2kv, the capillary temp of 350°C, and S-Lens RF Level of 60%. ChromaTOF software (V4.3x, LECO) was employed to perform peak extraction, baseline correction, and peak alignment based on mass spectrometry data; the peaks were matched with LECO/Fiehn Metabolomics Library database to identify and authenticate different metabolites.

The online platform MetaboAnalyst 5.0 was utilized to perform the statistical analysis and multivariate analysis of the metabolome dataset (https://www.metaboanalyst.ca/MetaboAnalyst/faces/home.xhtml). Principal component analysis (PCA) along with orthogonal projections to latent structures-discriminant analysis (OPLS-DA) was performed on the peaks extracted from all rumen samples of four treatments. The *q* value (FDR-adjusted *P*-value) <0.05 and variable importance projection (VIP) >1.0 were considered to be significantly different metabolites in the pairwise comparison. The differential metabolic pathways of the pairwise comparison were mainly analyzed through the pathway enrichment module in the online platform.

### Analysis of Correlations and Network Structure

Correlation analysis among rumen microbiota, metabolites, fermentation, and N metabolism was performed using Spearman's rank correlation, *q*-values were calculated using “Psych” R packages, *q*-values under 0.05 were considered as significant for correlation. The correlation heatmap and correlation network maps were visualized by “ggcorrplot” R package and Gephi software (version 0.9.2).

### Statistical Analysis

PROC MIXED procedure of SAS software (version 9.3, SAS Institute Inc., Cary, NC, US) was used for the data analysis. The treatment effects on ADG, DMI, ATTD, and N balance were estimated using the following model:


$${\text{Y}}_{\text{ijk}}=\mu {+\text{T}}_{\text{i}}{+\text{P}}_{\text{j}}+{\left(\text{T}\times \text{P}\right)}_{\text{ij}}{+\text{S}}_{\text{k }}+\varepsilon_{\text{ijk}}$$

Where *Y*_*ijk*_ is the dependent variable, μ is the mean, *T*_*i*_ is the fixed effect of treatments, *P*_*j*_ is the fixed effect of sampling period, (*T*×*P*)_*ij*_ is the fixed interaction between treatments and sampling period, *S*_*k*_ is the random effect of sheep, and ε_*ijk*_ is the residual variation. Data for the rumen α-diversity index were analyzed by PROC GLM procedure. Tukey's method was utilized for multiple comparisons. The threshold was declared at *P* < 0.05 and tendency at 0.05 ≤ *P* < 0.1.

## Results

### Feed Intake and Growth Performance

The results indicated that there was no significant difference between the feed intake and growth performance of each treatment (*P* > 0.05), but ADG and DMI were observed to be the highest in LPB, numerically (Table 2).

**Table 2:** Effects of SP (% of CP) in low-protein diets on growth performance in fattening *Hu* sheep (*n* = 8).

**Item**	**Treatments**				**SEM**	***P*-Value**
	**CON**	**LPA**	**LPB**	**LPC**		
Initial BW (kg)	40.56	40.53	40.80	39.59	0.837	0.964
Final BW (kg)	46.41	46.59	47.42	45.72	0.753	0.527
ADG (kg/d)	0.21	0.22	0.24	0.22	0.157	0.487
DMI (kg/d)	0.97	0.95	0.99	0.95	0.175	0.323
FCR	4.73	4.40	4.14	4.28	0.212	0.149

*BW, body weight; ADG, average daily gain; DMI, dry matter intake; FCR, feed conversion ratio. Treatments: CON is 16.7% CP based on nutritional requirements, CP of LPA, LPB, and LPC is decreased by ~10%, SP proportion (% of CP) 21.2, 25.9, and 29.4, respectively*.

### Apparent Total Tract Digestibility

As shown in Table 3, the DM and CP digestibility of the LPB were higher than CON and LPA (*P* < 0.05), organic matter (OM) digestibility was higher in the LPB (*P* < 0.05), whereas the digestibility of NDF in the LPA group was lower as compared with the LPB (*P* < 0.05).

**Table 3:** Effects of SP (% of CP) in low-protein diets on apparent total-tract digestibility (ATTD) of nutrients in fattening *Hu* sheep (*n* = 8).

**Item**	**Treatments**				**SEM**	***P*-Value**
	**CON**	**LPA**	**LPB**	**LPC**		
DM	67.33^ab^	62.57^b^	71.94^a^	64.84^ab^	0.931	0.008
OM	74.74^b^	72.66^b^	85.63^a^	73.27^b^	2.132	<0.001
CP	66.45^b^	66.85^b^	73.96^a^	69.81^ab^	2.032	0.044
NDF	50.84^bc^	47.34^c^	62.32^a^	52.91^ab^	3.021	0.045
ADF	37.98	34.82	41.57	40.30	4.584	0.545

*SP, Soluble protein; DM, dry matter; OM, organic matter; CP, crude protein; NDF, neutral detergent fiber; ADF, acid detergent fiber*.

a–c *values within a row with different superscripts differ significantly at p ≤ 0.05*.

*Treatments: CON is 16.7% CP based on nutritional requirements, CP of LPA, LPB, and LPC is decreased by ~10%, SP proportion (% of CP) 21.2, 25.9, and 29.4, respectively*.

### N Metabolism

The serum urea N (Supplementary Figure 1A) in low protein treatments was lower than CON (*P* < 0.05), while the urinary urea N (Supplementary Figure 1B) of LPB and LPC were lower than CON (*P* < 0.05).

As shown in Table 4, N intake in the low-protein treatments (LPA, LPB, and LPC) was lower than CON (*P* < 0.05); the fecal N excretion (% of N intake) of LPB and LPC was lower than CON and LPA (*P* < 0.05), whereas urinary N excretion (% of N intake) was decreased in LPB and LPC as compared with CON (*P* < 0.05). Moreover, N retention (% of N intake) and BVN in LPB and LPC were higher than CON and LPA (*P* < 0.05).

**Table 4:** Effects of SP (% of CP) in low-protein diets on nitrogen balance in fattening *Hu* sheep (*n* = 8).

**Item**	**Treatments**				**SEM**	***P*-Value**
	**CON**	**LPA**	**LPB**	**LPC**		
N intake (g/d)	25.90^a^	23.57^b^	24.66^b^	23.78^b^	0.113	<0.001
**Fecal N excretion**						
g/d	8.69^a^	7.81^a^	6.42^b^	7.18^ab^	0.164	<0.001
% of N intake	33.46^a^	33.15^a^	26.35^b^	30.19^b^	1.506	<0.001
**Urinary N excretion**						
g/d	9.07^a^	8.14^ab^	7.48^b^	7.11^b^	0.336	0.012
% of N intake	35.01^a^	34.26^ab^	30.34^b^	29.91^b^	0.953	0.032
**N retention**						
g/d	8.14^b^	7.61^b^	10.76^a^	9.50^a^	0.455	<0.001
% of N intake	31.44^b^	32.59^b^	43.62^a^	39.90^a^	1.063	<0.001
BVN (%)	47.31^b^	48.30^b^	58.98^a^	57.20^a^	1.051	<0.001

*BVN, biological value of N*.

a, b *values within a row with different superscripts differ significantly at P ≤ 0.05*.

*Treatments: CON is 16.7% CP based on nutritional requirements, CP of LPA, LPB, and LPC is decreased by ~10%, SP proportion (% of CP) 21.2, 25.9, and 29.4, respectively*.

### Rumen Fermentation

Rumen fermentation parameter results (Table 5) showed that NH_3_-N concentration in LPA and LPB was lower than CON (*P* < 0.05); the concentration of TVFA, acetate, and butanoate were all higher in CON (*P* < 0.05), and lower in LPA (*P* < 0.05) on the contrary. In addition, propanoate concentration in LPA was lower than CON and LPB (*P* < 0.05).

**Table 5:** Effect of low-protein diet with different SP (% of CP) on rumen fermentation of fattening *Hu* sheep (*n* = 6).

**Item**	**Treatments**				**SEM**	***P*-Value**
	**CON**	**LPA**	**LPB**	**LPC**		
pH	6.77	6.84	6.89	7.05	0.078	0.693
NH_3_-N (mg/dL)	10.51^a^	3.43^b^	4.40^b^	6.53^ab^	0.871	0.045
MCP (mg/mL)	1.86	1.29	1.38	1.15	0.157	0.441
**VFA, mM**						
TVFA	85.97^a^	44.76^c^	65.54^b^	66.27^b^	4.040	0.001
Acetate	58.26^a^	29.90^c^	43.90^b^	44.57^b^	2.994	0.003
Propionate	18.09^a^	9.56^c^	14.22^ab^	13.82^b^	0.866	0.001
Butyrate	6.22^a^	2.92^c^	4.54^b^	4.64^b^	0.395	0.021
Isobutyrate	1.14	0.85	0.96	1.01	0.070	0.573
Valerate	0.64	0.36	0.50	0.54	0.044	0.158
Isovalerate	1.63	1.17	1.43	1.70	0.146	0.615
A/P ratio	3.39	3.17	3.15	3.28	0.154	0.534

*MCP, Microbial crude protein; VFA, Volatile Fatty Acid; TVFA (total Volatile Fatty Acid), acetate + propionate + butyrate + valerate + isobutyrate + isovalerate; A/P ratio, acetate to propionate ratio*.

a–c *values within a row with different superscripts differ significantly at p ≤ 0.05*.

*Treatments: CON is 16.7% CP based on nutritional requirements, CP of LPA, LPB, and LPC is decreased by ~10%, SP proportion (% of CP) 21.2, 25.9, and 29.4, respectively*.

### Rumen Microbiome Composition and Taxonomic Differences

**Figure 1:**
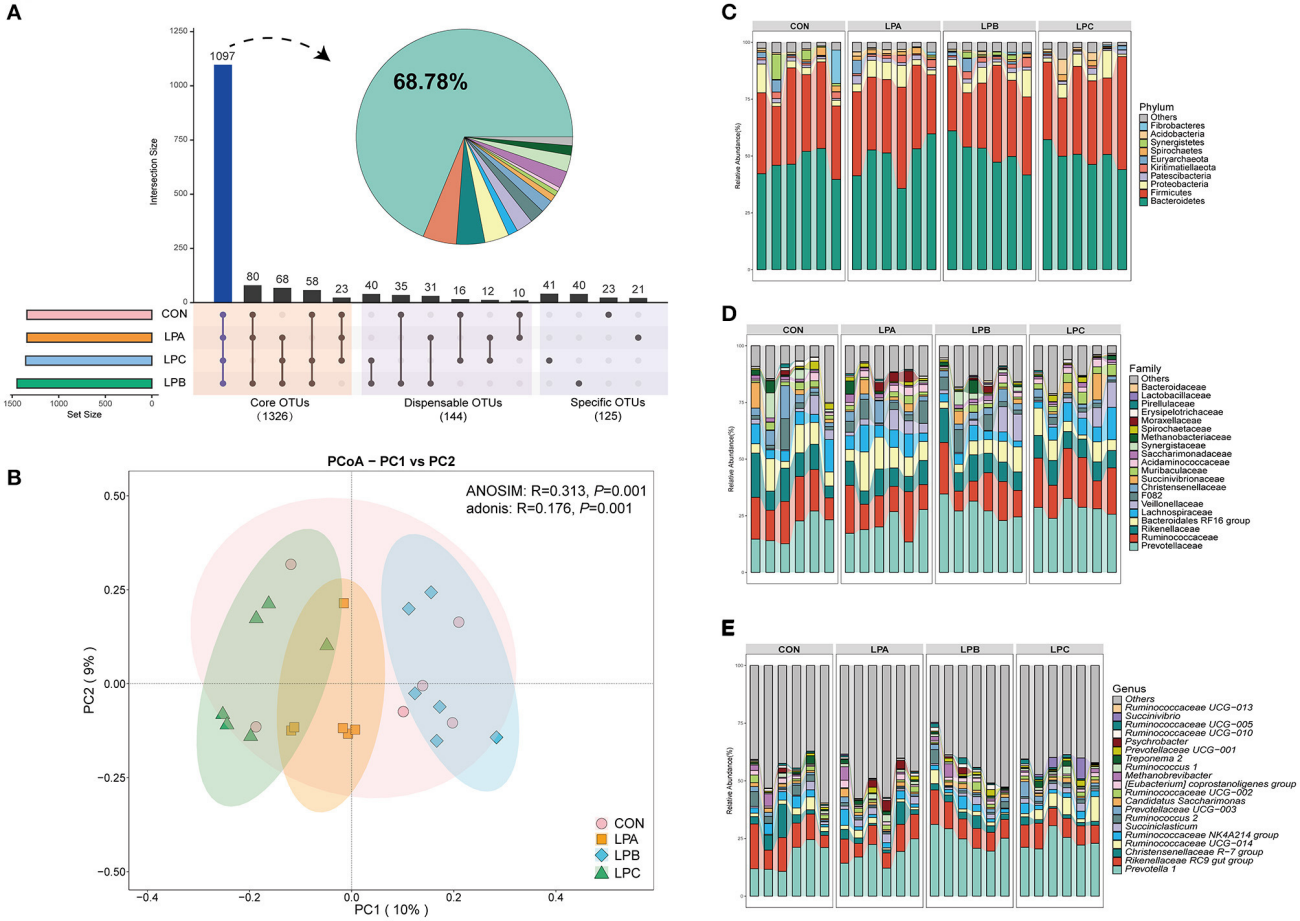
Bacterial community compositions in the rumen under four different treatments (*n* = 6). **(A)** The histograms show core operational taxonomic units (OTUs) (present in three or four treatments), dispensable OTUs (present in two treatments), and specific OTUs (present in one treatment). **(B)** Principal coordinates analysis (PCoA) revealed the separation of the microbial communities in the rumen among the four treatments based on the Bray–Curtis dissimilarity matrix. Bacterial compositions in the rumen of CON, LPA, LPB, and LPC treatments at the phylum **(C)**, family **(D)**, and genus **(E)** levels. Treatments: CON is 16.7% CP based on nutritional requirements, CP of LPA, LPB, and LPC is decreased by ~10%, SP proportion (% of CP) 21.2, 25.9, and 29.4, respectively.

In the rumen microbiome, a total of 933, 984 reads were obtained from the rumen samples, and an average of 49, 393 effective data were achieved after quality control, based on 97% sequence similarity, generating a total of 2, 242 OTUs (mean = 1, 263). The intersection numbers of OTU (Figure 1A) for the four groups were 1,097, which accounted for 68.78% of the total OTU number; the specific OTU numbers for CON, LPA, LPB, and LPC were 23, 21, 40, and 41 respectively. In Supplementary Table 2, the LPB group increased the ACE index and Chao 1 index (*P* < 0.05), while there was no difference in the bacterial community diversity (Shannon and Simpson) and coverage (*P* > 0.05). The PCoA results (Figure 1B) showed that the bacterial communities from the four treatments were isolated from one another based on Bray–Curtis dissimilarity matrix (ANOSIM: *P* = 0.001; Adonis: *P* = 0.001).

**Figure 2:**
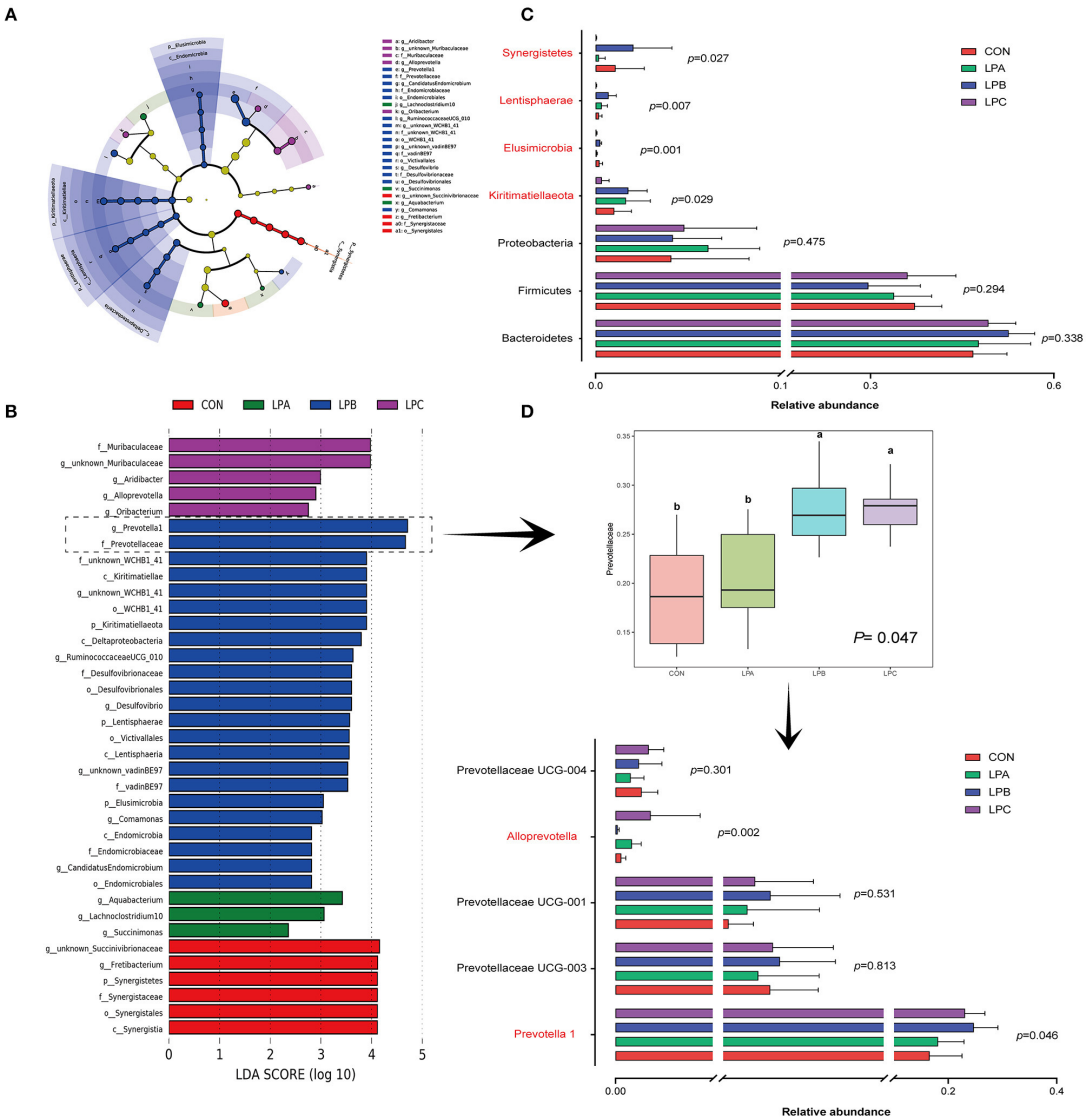
Differential microbial enrichment analysis among four treatments (*n* = 6). **(A)** Dendrogram indicating that multiple taxa are differentially enriched in the rumen samples of four treatments. The taxonomic groups are differentially enriched in the corresponding groups: red (CON), green (LPA), blue (LPB), and purple (LPC). **(B)** Linear discriminant analysis (LDA) scores of the microbial OTUs in the rumen of *Hu* sheep fed with CON, LPA, LPB, and LPC diets, LDA score > 2 was marked. **(C)** Bar chart of differential microbial phylum levels in the rumen of four treatments. **(D)** Relative abundance of Prevotellaceae and the top-five abundance genus belong to Prevotellaceae. Treatments: CON is 16.7% CP based on nutritional requirements, CP of LPA, LPB, and LPC is decreased by ~10%, SP proportion (% of CP) 21.2, 25.9, and 29.4, respectively.

At the phylum level (Figure 1C), bacteroidetes and firmicutes were the dominant phyla of four treatments, it is observed from the comparison of four treatments (Figures 2A–C) that Kiritimatiellaeota, Elusimicrobia, and Lentisphaerae were enriched in LPB and Synergistetes was enriched in CON (LDA>3, *P* < 0.05). At the family level (Figure 1D), Prevotellaceae and Ruminococcaceae were the dominant families, while LPB and LPC increased the abundance of Prevotellaceae (LDA>4, *P* < 0.05), especially in LPB (Figures 2B,D). At the genus level (Figure 1E), *Prevotella_1* and *Rikenellaceae_RC9_gut_group* were the dominant genus, *Prevotella_1* and *Ruminococcaceae UCG-010* were enriched in LPB (LDA > 3, *P* < 0.05, Figure 2B), which were also the top-20 dominant genus.

### Rumen Metabolite Composition, Differential Metabolites, and Pathway Enrichment

**Figure 3:**
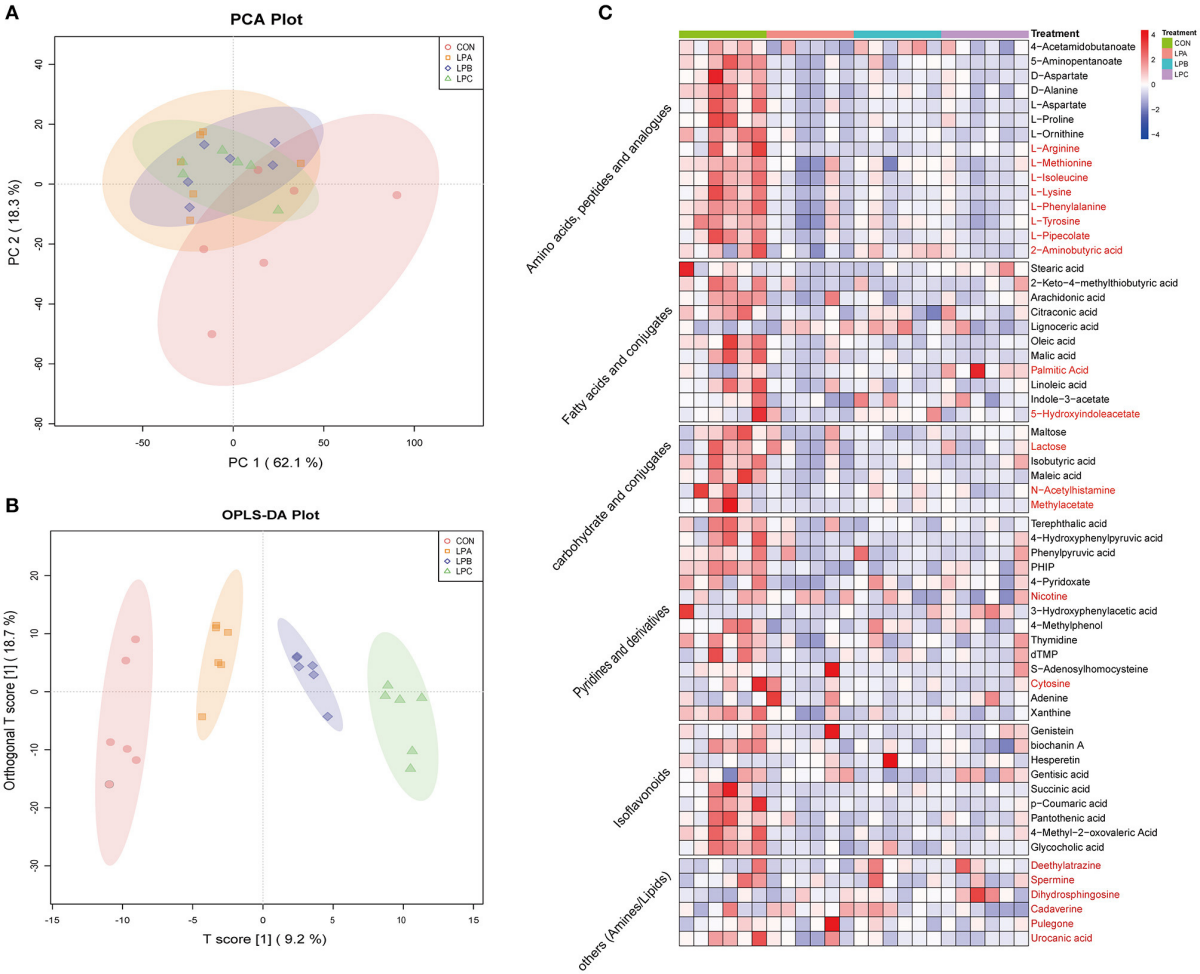
Differences in rumen metabolites of *Hu* sheep fed with CON, LPA, LPB, and LPC diets (*n* = 6). **(A)** Principal Component Analysis (PCA) based on rumen metabolites of four treatments. **(B)** Orthogonal projections to latent structures-discriminant analysis (OPLS-DA) plot based on a liquid chromatograph-mass spectrometer (LC-MS) analysis of four treatments. **(C)** Heatmap showed significantly different rumen metabolites among four treatments, metabolites in black font were detected in positive mode, and red were detected in negative mode. Treatments: CON is 16.7% CP based on nutritional requirements, CP of LPA, LPB, and LPC is decreased by ~10%, SP proportion (% of CP) 21.2, 25.9, and 29.4, respectively.

Based on the LC-MS system, 556 and 653 peaks were retained after preprocessing the original data of the positive and negative ion modes, with 263 compounds were identified and quantified finally. PCA score plot was listed in Figure 3A, which showed the metabolite composition. Meanwhile, the OPLS-DA plot (Figure 3B) showed that the four treatments are clearly separated. The OPLS-DA, score results and corresponding validation plots (Supplementary Figures 2A–F) were drawn to assess the model quality control parameters, all samples in the score plots of the six comparisons are within the 95% confidence interval (Hotelling's T-squared ellipse), and the corresponding *R*^2^*Y* values were all >0.99, indicating the satisfactory effectiveness of the model and can be used for subsequent difference analysis.

A total of 63 metabolites were obtained significantly different (VIP > 1.0 and *q* < 0.05; Figure 3C) from the comparisons of CON vs. LPA, CON vs. LPB, CON vs. LPC, LPA vs. LPB, LPA vs. LPC, and LPB vs. LPC, which were mainly classified into amino acids, peptides, fatty acids, carbohydrates, pyrimidines, and isoflavonoids. Among them, 14 metabolites (Supplementary Table 3) were mainly detected with significant differences from the pairwise comparison of the last three groups (LPA, LPB, and LPC), and 4-Pyridoxate and succinic acid significantly increased in LPB and LPC (*q* < 0.05).

**Figure 4:**
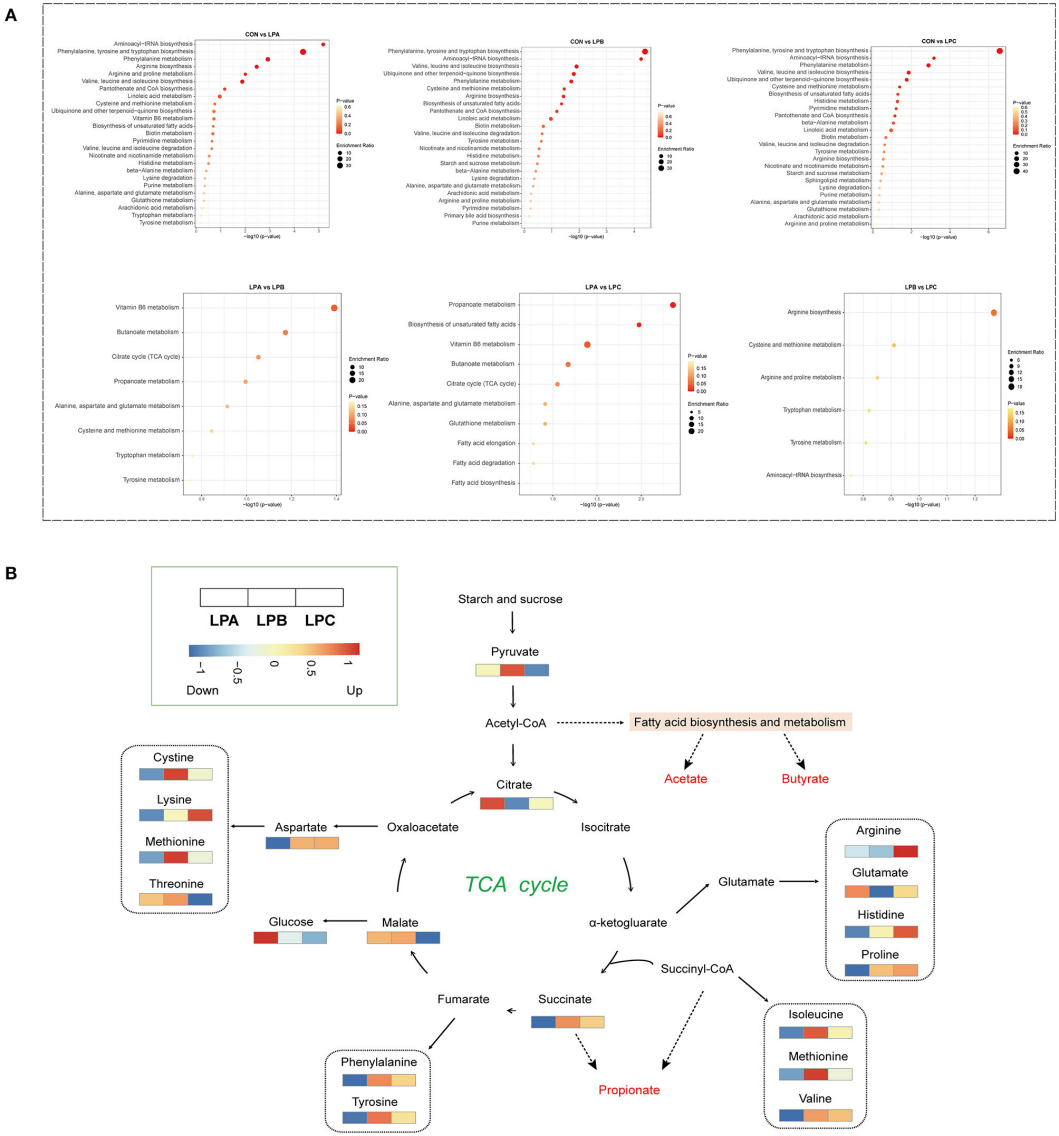
Enrichment analysis of metabolic pathways of differential rumen metabolites among four treatments. **(A)** Kyoto Encyclopedia of Genes and Genomes (KEGG) pathway enrichment analysis performed using the significantly different rumen metabolites based on the pairwise comparison (CON vs. LPA, CON vs. LPB, CON vs. LPC, LPA vs. LPB, LPA vs. LPC, and LPB vs. LPC). **(B)** Schematic diagram of the main different metabolic pathways of LPA, LPB, and LPC; heatmap showing the relative abundance of significantly different metabolites. Treatments: CON is 16.7% CP based on nutritional requirements, CP of LPA, LPB, and LPC is decreased by ~10%, SP proportion (% of CP) 21.2, 25.9, and 29.4, respectively.

Metabolic pathway enrichment analysis (Figure 4A) based on significantly different rumen metabolites revealed that low-protein diet treatment changed a total of 28 metabolic pathways in the rumen, with “Aminoacyl-tRNA biosynthesis,” “Phenylalanine, tyrosine, and tryptophan biosynthesis,” “phenylalanine metabolism,” and “valine, leucine, and isoleucine biosynthesis” all being the significant pathways (*P* < 0.05) in three low-protein diets (LPA, LPB, and LPC). In addition, with the increase in the SP proportion in low-protein diets, fatty acid metabolism was significantly changed, especially “propanoate metabolism,” “butanoate metabolism” and “vitamin B6 metabolism”. As shown in Figure 4B, a schematic diagram of the citrate cycle (TCA cycle) revealed the relationship between fatty acid metabolism and amino acid metabolites among different SP treatments in low-protein diets.

### Correlations of Rumen Microbiome, Metabolome, and Phenotype

**Figure 5:**
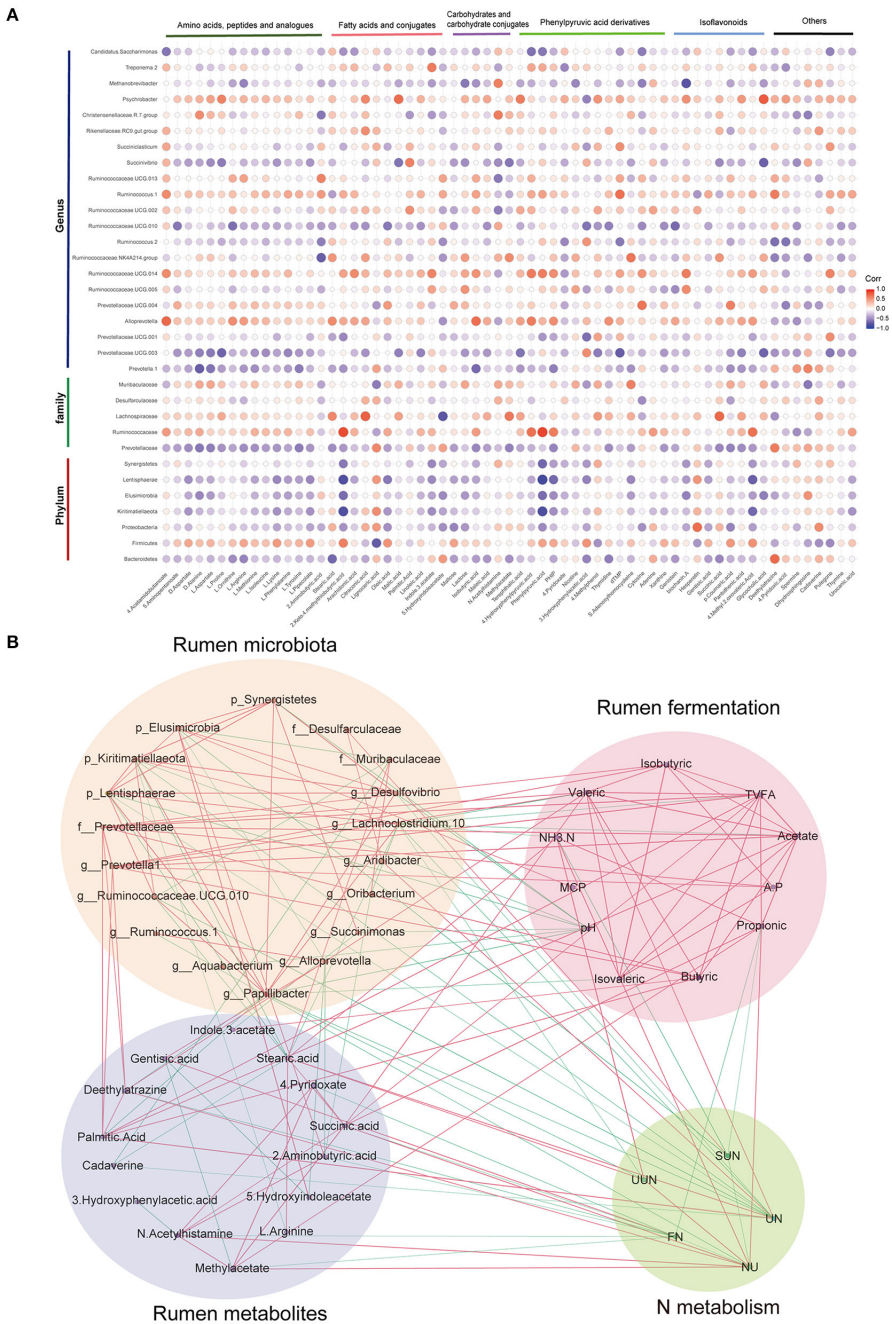
Interaction between rumen microbiota, metabolites, fermentation, and N metabolism. **(A)** Spearman's rank correlations between rumen microbiota and rumen microbial metabolites; the blue circle represents negative correlations and red circle represents positive correlations. **(B)** Spearman's correlation network showing relationships among rumen microbiota, metabolites, fermentation, and N metabolism, which significantly differ in LPA, LPB, and LPC. Only strong correlations (|*r*| > 0.5, *q* < 0.05) are shown in the correlation networks. FN: fecal nitrogen excretion; UN: urine nitrogen excretion; SUN: Serum urea nitrogen; UUN: Urinary urea nitrogen; NU: nitrogen utilization.

Spearman's rank correlations between the different rumen microbiota and rumen metabolites (Figure 5A) revealed that Kiritimatiellaeota, Elusimicrobia, Lentisphaerae, Prevotellaceae, *Prevotella 1*, and *Ruminococcaceae UCG-010*, which are enriched in LPB, showed negative correlations with amino acids, peptides, and analogs. Spearman's rank correlation network (Figure 5B) mainly showed the close and complex relationship among rumen microbiota, metabolites, fermentation, and N metabolism, which are mainly enriched in three low-protein diets with different SP treatments (LPA, LPB, and LPC). Among them, the interaction of rumen microbiota was the most complicated, where either the rumen microbiota or the metabolites, were connected with phenotype to some extent. Among the N metabolism indices, urine N excretion had the largest number of connections with the rumen microbiota, metabolites, and fermentation (11 connections in total), followed by N utilization (10) and fecal N excretion (8).

## Discussion

In the current study, dietary CP level was reduced by ~10%, while it did not affect the growth performance of *Hu* sheep, which was consistent with the study by Zhu et al. (37), showing that by reducing the CP by ~10% (from 14.8 to 13.4%), ADG and DMI were not affected. Meanwhile, thestudies of Lv et al. (38) on lambs (CP 15.7% vs. 11.8%) and Arias et al. (39) on Heifers (CP 15.1% vs. 12.7%) also got similar results. However, studies have reported that a low-protein diet could inhibit the growth performance of Hainan black goats (CP 17% vs. 15%) or lambs (CP 20.8% vs. 16.4%) (40, 41), which is mainly related to the species of animal, age, and environment (42). While changing the SP proportions in low-protein diets, ADG, DMI, and FCR did not differ among treatments, which was also consistent with the results of Majdoub et al. (17). However, the age choice of sheep (6-month-old) might be an important factor affecting the growth performance in this study, ADG has a weak response to the end of growth stage compared with the rapid growth period, which might lead to no difference in the growth performance.

A low-protein diet had a great influence on the nutrient digestibility of monogastric animals and ruminants (43, 44). The ATTD was increased in LPB, which might indicate that higher CP digestibility improved the activity of cellulose-degrading bacteria and starch-degrading bacteria, thereby increasing the OM and PDF digestibility. It has been shown that LPB also increased the bacterial richness (Supplementary Table 3), which has been proved to be a key factor affecting the nutrient digestibility in ruminants (45, 46), indicating the potential reason for the increase of digestion and absorption. A reasonable explanation for the increase in the bacterial richness might be that LPB contained both soybean meal and urea compared to LPA and LPC. A previous study (47) reported that when soybean meal and urea were supplied together, the digestibility was improved compared with the usage of only one, which indicated that SP content provided by the appropriate ratio of soybean meal and urea might promote the balance between energy and N, resulting in the proliferation of microorganisms and in the increase of ATTD. In addition, using urea to alter dietary SP fractions may lead to low CP utilization (48), but it did not seem to happen in our research, which is mainly related to the addition level.

Dietary proteins are degraded to NH_3_-N, amino acids and peptides in the rumen and the excess ammonia greatly increased the serum urea N and urine N (49, 50). It has been reported in most of the studies that by feeding ruminants with a low-protein diet, serum urea N, and ruminal NH_3_-N is reduced (51, 52). Our study was also consistent with these findings. Wilson et al. (16) showed that with the increase of dietary SP (%CP) from 34.4 to 44.9, the plasma urea N was also increased. In the present study, with the increase of SP (%CP), there was no obvious difference observed in serum urea N. It might be the reason that the previous studies had a higher SP ratio as compared to our study, where excessive SP led to the increase of serum urea N (53). Moreover, ruminal NH_3_-N increased numerically (~3.43–6.53 mg/100 mL), which is also similar to the results as reported by Wilson et al. (16). Du et al. (54) found that replacing the protein source with a high degradation rate in the concentrate with a protein source of low degradation rate in legumes can reduce the ruminal NH_3_-N and blood urea N. Although there is usually a strong positive correlation between rumen NH_3_-N and blood urea N (55), it appeared that NH_3_-N concentrations were not high enough to elevate the serum urea N in our study.

In this study, we found that when the SP proportion of low-protein diet was 25.9 and 29.4, the fecal N excretion (% of N intake) was significantly reduced as compared with CON, which was mainly related to higher CP digestibility and rumen microbial activity. Urinary N excretion was also decreased in these two treatments and showed the same changing trend as the output of urea N in urine, while Ding et al. (27) also reported that they had a strong positive correlation. Furthermore, Ahvenjarvi and Huhtanen (56) reported that with the increase of urea-N infusion level (0, 17, 33, 49, and 66 g/d) in the rumen of dairy cows, the N excretion of fecal and urine increased linearly. N retention (% of N intake) increased in three low-protein diets, which was inconsistent with the studies of Zhu et al. (37) and Hynes et al. (57), indicating that to some extent, reducing dietary protein levels can improve N efficiency. Aitchison et al. (58) found that N utilization in lactating dairy cows decreased with the increasing soluble N proportion of the diets (35.4, 40.6, 43.3, and 47.5% CP). Majdoub et al. (17) also got similar results, showing that regardless of feeding dairy cows with high or low protein diets (CP 15.3% vs. 12.2%), the retained N was all low in high SP proportion (22 vs. 44% CP). Interestingly, in the present study, N retention of low-protein diets ranked as follows: LPB > LPC > LPA, with SP proportion increased, it reached the peak at 25.9, which seemed to reveal that this proportion may be the optimum, as it indeed increased the rumen microbial activity to utilize ammonia. Moreover, the SP proportion in previous studies seemed to be relatively high, and majority of SP substrate was degraded too fast to be fully utilized by rumen microorganisms, resulting in a decrease in N efficiency (59).

Similar to several previous studies evaluating the rumen microbiome (38), bacteroidetes, firmicutes, and proteobacteria were the dominant phyla, which were key participants in the degradation and fermentation of most feed biopolymers. Notably, at the family and genus level, Prevotellaceae and *Prevotella1* are both enriched in LPB. *Prevotella* genus utilizes starch and protein to produce succinate and acetate, which is one of the most abundant core genera in the rumen and plays an important role in the biosynthesis of VFAs (60, 61). In this study, *Prevotella1* of LPB and LPC was higher than LPA and Kiritimatiellaeota is also enriched in LPB. Kiritimatiellaeota participates in arginine and fatty acid biosynthesis, which can efficiently use N to produce energy (62). In addition, *Lachnoclostridium 10* was enriched in LPA, while it is reported that *Lachnoclostridium* can be used as biomarkers diagnosed with colorectal adenoma and colon cancer (63), which may lead to lower VFA production, but the mechanism of *Lachnoclostridium* regulating nutrient transport in ruminant is still unclear. Interestingly, Synergistetes was enriched in CON, while the genomic features of Synergistetes include amino acids, peptides, and protein transport and metabolism (64); however, Zhang et al. (65) found that feeding a high-protein diet reduced the abundance of Synergistete in the rumen of calves. Whereas, the relative abundance was very low in this study (0.001–0.02%), which needs to be further studied in the relationship of N utilization in the rumen.

Rumen metabolomics showed that low-protein diets reduced the levels of 15 amino acids (D-aspartate, D-alanine, L-phenylalanine, etc.), which were mainly derived from the dietary protein and microbial proteins degraded by rumen microbiota. Previous studies have demonstrated that rumen microorganisms can use acetate, propionate, or other substances as a carbon source, and ammonia and other N-containing compounds as a N source for *de novo* synthesis of amino acids (66, 67). Therefore, the high concentration of propionate and butyrate in CON may partially promote the biosynthesis of amino acids. The pathway enrichment observed in this study included aminoacyl-tRNA biosynthesis; phenylalanine, tyrosine, and tryptophan biosynthesis; arginine biosynthesis; valine, leucine, and isoleucine biosynthesis, which also confirmed the above conclusion. Therefore, higher dietary protein levels may cause excessive fermentation in the rumen, and excessive N will be excreted to the environment.

Importantly, with increased SP levels in the low-protein diets, both 4-Pyridoxate and Succinate were increased in LPB and LPC. The corresponding pathway enrichments include propanoate metabolism, butanoate metabolism, biosynthesis of unsaturated fatty acids, and citrate cycle (TCA cycle). It was previously reported that in the rumen of animals with high feed efficiency, VFA concentration, such as butyrate and propionate is higher and the methane emission is lower (68). The higher butyrate and propionate concentrations and enrichment pathways in LPB and LPC of this study seemed to imply higher feed efficiency. In future studies, we need to measure methane emissions to verify our speculations. Furthermore, it is worth noticing that vitamin B6 metabolism is strengthened with increased SP, whereas vitamin B6 (a coenzyme factor) is closely related to amino acid metabolism (69). Many recent studies have reported that supplementation of vitamin B compounds can increase dairy cows' production and milk protein (69, 70). Therefore, the appropriate dietary SP levels (LPB and LPC) in this study may regulate rumen microbes to produce more vitamin B, which may improve N efficiency.

Diet-microbiome interaction greatly affects feed efficiency (71). Here, adjusting SP levels in low-protein diets affect the rumen microbiome and metabolites, which in turn affects rumen fermentation and N utilization. Xue et al. (60) reported that *Prevotella* species may affect the host's amino acid metabolism. Meanwhile, comparative genomes have also confirmed their important role in N metabolism (72). In our study, *Prevotella* was negatively correlated with amino acids, which also indicated that *Prevotella* promotes the metabolism of amino acids. Moreover, the complex network connection among microorganisms, metabolites, and rumen fermentation directly affects the body's N utilization. However, diets with high SP may be unfavorable to N metabolism, such as urea (100% soluble), high dietary urea addition level would inhibit the growth of microorganisms in the rumen and might affect rumen fermentation and nutrient utilization (73).

## Conclusions

**Figure 6:**
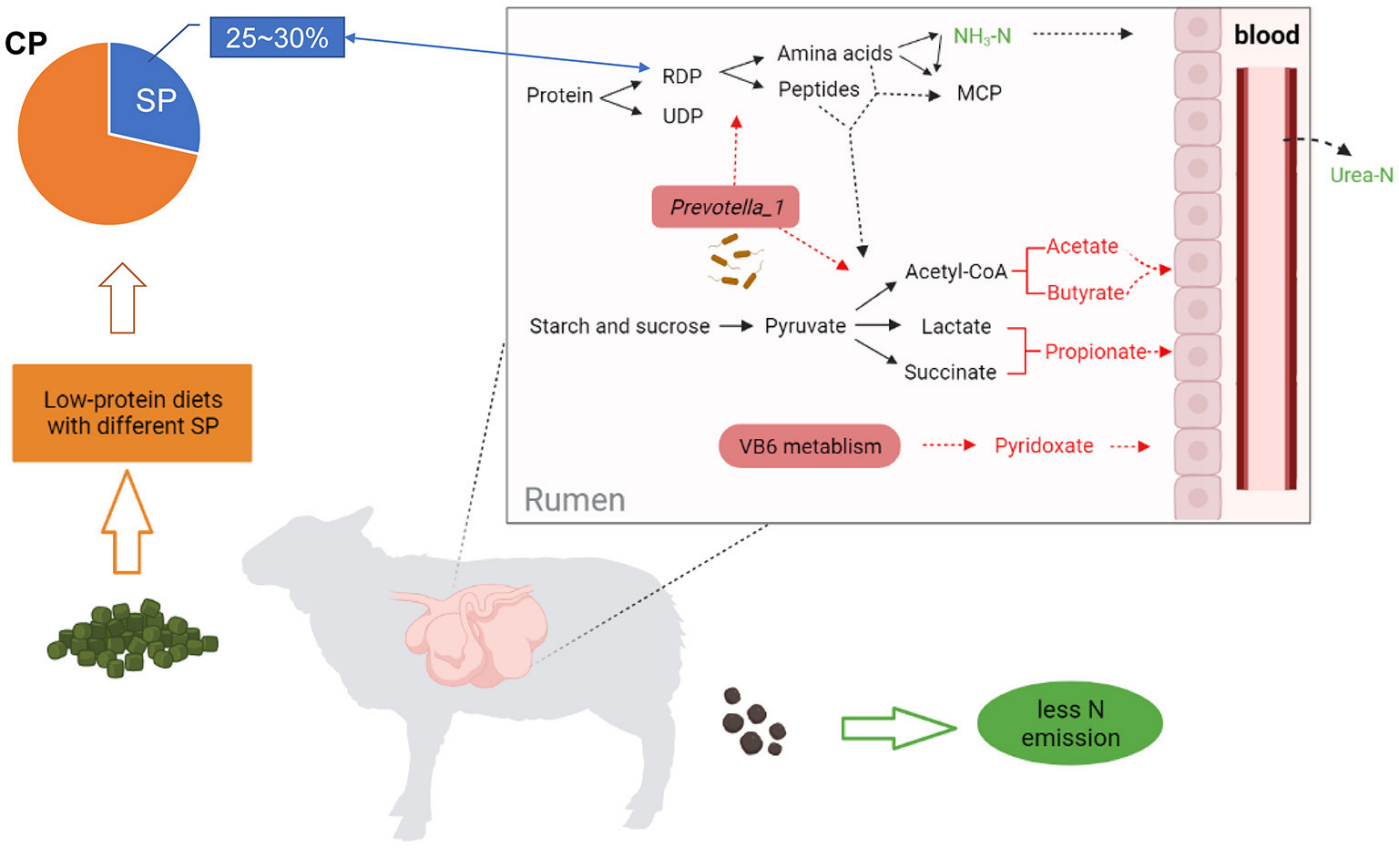
A holistic view of diet-microbiome co-interaction patterns in remodeling of rumen N metabolism during adaptation to a low-protein diet with SP (about 25~30% of CP) in a *Hu* sheep model. Red fonts and arrows represent up-regulated, green represent down-regulated. CP, crude protein; SP, soluble protein; RDP, rumen degradable protein; UDP, rumen undegradable protein; MCP, microbial crude protein.

Our study concluded that decreasing dietary CP by ~10% can reduce the serum urea N and ruminal NH_3_-N without affecting the growth performance of fattening *Hu* sheep (BW 40 kg, ADG 200–250 g/d). Meanwhile, when feeding sheep (ruminants) with decreasing dietary CP content, the consideration of dietary SP level (25–30%) seems to be an important variable to avoid excessive N emissions or optimize animal performance, because SP levels of 25.9 and 29.4% indeed improved N efficiency in this study. More specifically, these levels increased the ruminal microbiota richness and the abundance of the *Prevotella_1* genus, which upregulated the biosynthesis of unsaturated fatty acids and vitamin B6 pathway, resulting in an increased VFA production as well as decreased urea-N in serum and urine (Figure 6). These findings could help us understand the role of different SP levels in the regulation of rumen microbial metabolism and N efficiency. Besides, the choice of CP source is also worth considering, appropriate dietary ratios of different CP sources (e.g., soybean meal) may improve digestibility. In future, we need to conduct more experiments on dietary with multiple CP sources to enrich our understanding of SP in ruminant production, and the mechanism of diet-microbiome-host interaction about SP level in low-protein diets feeding sheep requires deeper molecular research to reveal.

## Data Availability Statement

The datasets presented in this study can be found in online repositories. The names of the repository/repositories and accession number(s) can be found below: NCBI SRA BioProject, accession no: PRJNA780247.

## Ethics Statement

The animal study was reviewed and approved by Animal Welfare Committee of Yangzhou Veterinarians of the Agriculture Ministry of China (Yangzhou, China, license No. syxk (Su)2019-0029). Written informed consent was obtained from the owners for the participation of their animals in this study.

## Author Contributions

ZZ and MW: conceived and designed the study. ZZ, SS, RD, YL, ZL, CL, YC, RQ, and PG: conducted the experiment. ZZ, YL, ZL, and CL: analyzed the animal data. ZZ: performed the bioinformatics analysis and visualization and drafted the manuscript. ZZ, KS, QY, and MW: contributed to the writing of the manuscript. All the authors revised and approved the submitted version of the manuscript.

## Funding

This research was funded by the National 13th Five-Year Plan Key Research and Development Program (2018YFD0502100, 2017YFD0800200), the key program of State Key Laboratory of Sheep Genetic Improvement and Healthy Production (2021ZD07; 2021ZD01), and the Priority Academic Program Development of Jiangsu Higher Education Institutions (PAPD), P.R. China.

## Conflict of Interest

The authors declare that the research was conducted in the absence of any commercial or financial relationships that could be construed as a potential conflict of interest.

## Publisher's Note

All claims expressed in this article are solely those of the authors and do not necessarily represent those of their affiliated organizations, or those of the publisher, the editors and the reviewers. Any product that may be evaluated in this article, or claim that may be made by its manufacturer, is not guaranteed or endorsed by the publisher.

## Supplementary Material

Supplementary Figure 1 Effects of SP (%CP) in low-protein diets on urea-N in serum **(A)** and urine **(B)** of fattening *Hu* sheep (*n* = 8). ^a,b^Box plot with different superscripts differ significantly at *p* ≤ 0.05. Treatments: CON is 16.7% CP based on nutritional requirements, CP of LPA, LPB and LPC is decreased by ~10%, SP proportion (% of CP) 21.2, 25.9 and 29.4, respectively.

Supplementary Figure 2 OPLS-DA score plots and corresponding validation plots derived from the LC-MS metabolite profiles of rumen samples for sheep fed low protein diet with different SP. **(A)** CON vs. LPA, **(B)** CON vs. LPB, **(C)** CON vs. LPC, **(D)** LPA vs. LPB, **(E)** LPA vs. LPC, **(F)** LPB vs. LPC. Treatments: CON is 16.7% CP based on nutritional requirements, CP of LPA, LPB and LPC is decreased by ~10%, SP proportion (% of CP) 21.2, 25.9, and 29.4, respectively.

Supplementary Table 1 Protein fractions of the feed samples.

Supplementary Table 2 Effect of low-protein diet with different SP on bacterial richness, diversity and coverage (*n* = 6).

Supplementary Table 3 Different metabolites content in the rumen of sheep fed low protein diet with different SP (LPA, LPB and LPC) (n=6).
