# Supplementary figures and images for: Altering Dietary Soluble Protein Levels With Decreasing Crude Protein May Be a Potential Strategy to Improve Nitrogen Efficiency in Hu Sheep Based on Rumen Microbiome and Metabolomics
Source: Front Nutr. 2022 Jan 18;8:815358. doi: 10.3389/fnut.2021.815358 (PMC8804502; doi:10.3389/fnut.2021.815358)

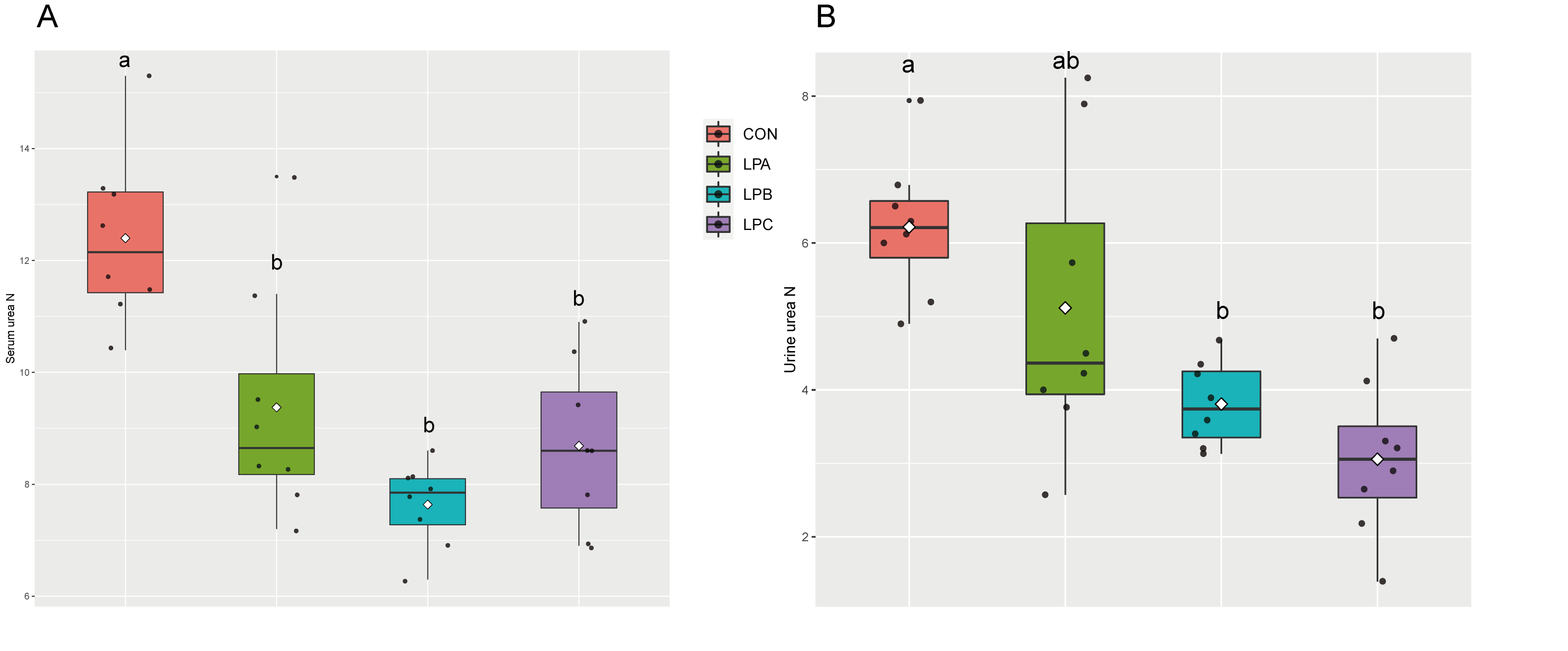

Supplement: Supplementary Figure 1 — Effects of SP (%CP) in low-protein diets on urea-N in serum (A) and urine (B) of fattening Hu sheep (n = 8). a,bBox plot with different superscripts differ significantly at p ≤ 0.05. Treatments: CON is 16.7% CP based on nutritional requirements, CP of LPA, LPB and LPC is decreased by ~10%, SP proportion (% of CP) 21.2, 25.9 and 29.4, respectively. [file Image_1.TIF]

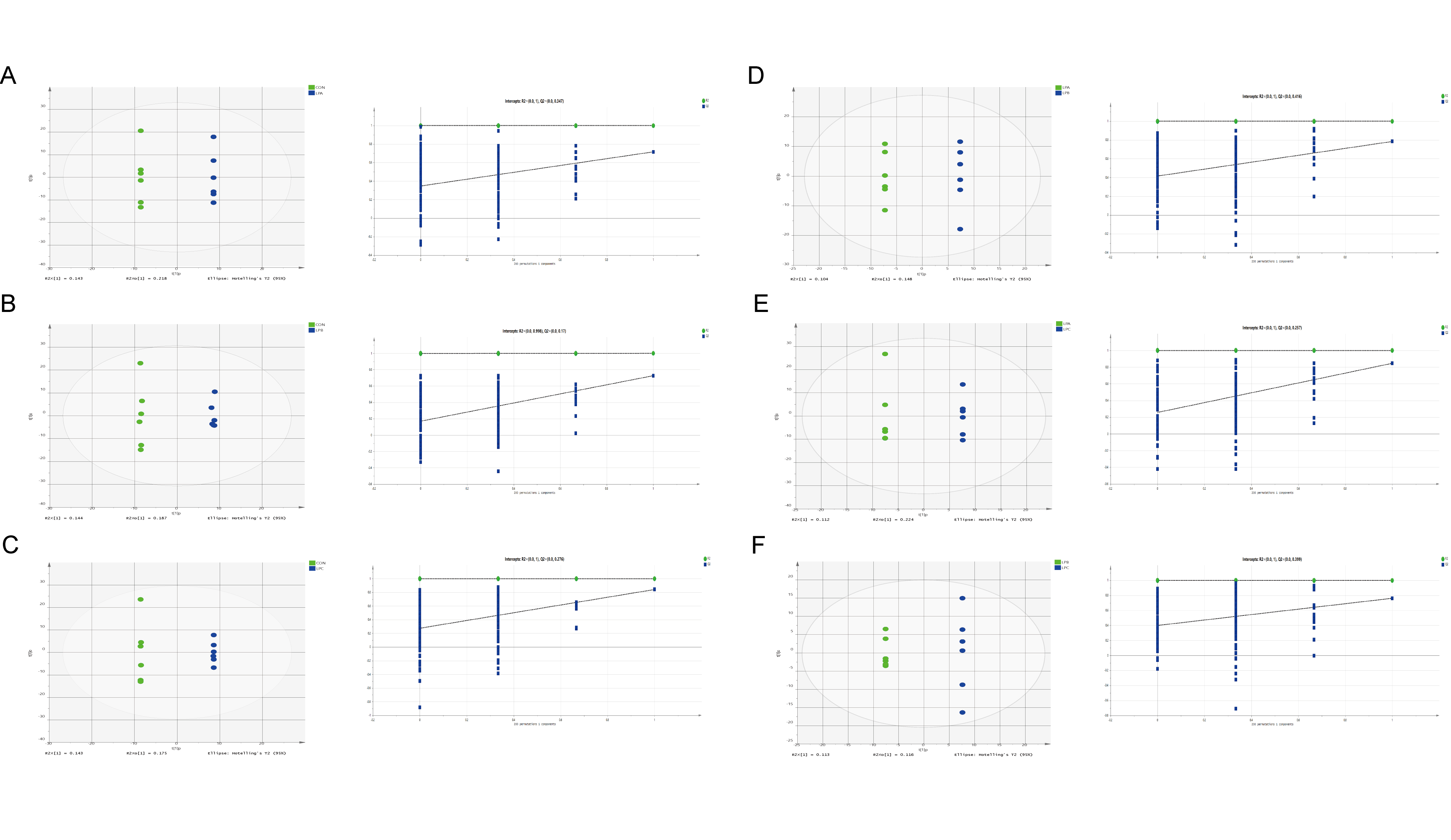

Supplement: Supplementary Figure 2 — OPLS-DA score plots and corresponding validation plots derived from the LC-MS metabolite profiles of rumen samples for sheep fed low protein diet with different SP. (A) CON vs. LPA, (B) CON vs. LPB, (C) CON vs. LPC, (D) LPA vs. LPB, (E) LPA vs. LPC, (F) LPB vs. LPC. Treatments: CON is 16.7% CP based on nutritional requirements, CP of LPA, LPB and LPC is decreased by ~10%, SP proportion (% of CP) 21.2, 25.9, and 29.4, respectively. [file Image_2.TIF]
