# Supplementary material for: Altering Dietary Soluble Protein Levels With Decreasing Crude Protein May Be a Potential Strategy to Improve Nitrogen Efficiency in Hu Sheep Based on Rumen Microbiome and Metabolomics
Source: Front Nutr. 2022 Jan 18;8:815358. doi: 10.3389/fnut.2021.815358 (PMC8804502; doi:10.3389/fnut.2021.815358)
Supplement: Supplementary Table 1 — Protein fractions of the feed samples. [file Table_1.DOCX]

**Table S1.** Protein fractions of the feed samples.

| Items | Protein fractions (Mean, n=3) | | |
| --- | --- | --- | --- |
|  | CP^1^ % | SP^2^ (%CP) | ISP^3^ (%CP) |
| Mixed silage | 19.32 | 20.1 | 79.9 |
| Corn | 8 | 24.3 | 75.7 |
| Soybean meal | 46.9 | 35.9 | 64.1 |
| Wheat bran | 15.4 | 33.7 | 66.3 |
| Corn protein meal | 65.5 | 6.4 | 93.6 |
| Urea | 281 | 100 | 0 |

^1^CP = crude protein;

^2^SP = soluble protein;

^3^ISP = insoluble protein.
