# Supplementary material for: Altering Dietary Soluble Protein Levels With Decreasing Crude Protein May Be a Potential Strategy to Improve Nitrogen Efficiency in Hu Sheep Based on Rumen Microbiome and Metabolomics
Source: Front Nutr. 2022 Jan 18;8:815358. doi: 10.3389/fnut.2021.815358 (PMC8804502; doi:10.3389/fnut.2021.815358)
Supplement: Supplementary Table 2 — Effect of low-protein diet with different SP on bacterial richness, diversity and coverage (n = 6). [file Table_2.docx]

**Table S2**. Effect of low-protein diet with different SP on bacterial richness, diversity and coverage (n=6).

| Item | Treatments | | | | SEM^1^ | *P*-value |
| --- | --- | --- | --- | --- | --- | --- |
|  | CON | LPA | LPB | LPC |  |  |
| Community richness | | | | | | |
| ACE_index | 1404.20^b^ | 1429.94 ^b^ | 1611.23^a^ | 1457.68 ^b^ | 30.363 | 0.040 |
| Chao1_index | 1411.16 ^b^ | 1419.57 ^b^ | 1588.92^a^ | 1479.98 ^b^ | 30.342 | 0.031 |
| Community diversity | | | | | | |
| Shannon_index | 5.01 | 5.04 | 5.48 | 5.23 | 0.092 | 0.239 |
| Simpson_index | 0.97 | 0.98 | 0.98 | 0.98 | 0.002 | 0.598 |
| Community coverage | | | | | | |
| Goods_coverage | 0.9959 | 0.9959 | 0.9957 | 0.9961 | 0.000 | 0.696 |

^1^ SEM, standard error of the mean

Treatments: CON is 16.7% CP based on nutritional requirements, CP of LPA, LPB and LPC is decreased by ~10%, SP proportion (% of CP) 21.2, 25.9 and 29.4 respectively.
