# Supplementary material for: Altering Dietary Soluble Protein Levels With Decreasing Crude Protein May Be a Potential Strategy to Improve Nitrogen Efficiency in Hu Sheep Based on Rumen Microbiome and Metabolomics
Source: Front Nutr. 2022 Jan 18;8:815358. doi: 10.3389/fnut.2021.815358 (PMC8804502; doi:10.3389/fnut.2021.815358)
Supplement: Supplementary Table 3 — Different metabolites content in the rumen of sheep fed low protein diet with different SP (LPA, LPB and LPC) (n=6). [file Table_3.docx]

**Table S3**. Different metabolites content in the rumen of sheep fed low protein diet with different SP (LPA, LPB and LPC) (n=6).

| LPA vs LPB | | | | | | |
| --- | --- | --- | --- | --- | --- | --- |
| Metabolites | LPA | LPB | VIP ^1^ | *q*-value | Log_2_FC ^2^ | Trend |
| Indole-3-lactic acid | 0.05 | 0.12 | 1.849 | 0.013 | 1.131 | up |
| 4-Pyridoxate | 1.20 | 2.33 | 1.776 | 0.022 | 0.955 | up |
| Gentisic acid | 1.69 | 0.87 | 1.907 | 0.012 | -0.950 | down |
| Succinic acid | 0.73 | 2.40 | 1.736 | 0.029 | 1.722 | up |
| 2-Aminobutyric acid | 0.01 | 0.02 | 1.836 | 0.004 | 0.897 | up |
| Atrazine-desethyl | 0.00 | 0.01 | 1.560 | 0.045 | 1.382 | up |
| N-Acetylhistamine | 0.08 | 0.16 | 1.651 | 0.023 | 0.995 | up |
| LPA vs LPC | | | | | | |
| Metabolites | LPA | LPC | VIP | *q*-value | Log_2_FC | Trend |
| 3-Hydroxyphenylacetic acid | 0.22 | 0.91 | 1.568 | 0.035 | 2.019 | up |
| 4-Pyridoxate | 1.20 | 2.27 | 1.480 | 0.039 | 0.915 | up |
| Methylacetate | 0.02 | 0.03 | 1.589 | 0.015 | 0.956 | up |
| Stearic acid | 1.45 | 8.43 | 1.571 | 0.034 | 2.540 | up |
| Succinic acid | 0.73 | 2.13 | 1.827 | 0.003 | 1.555 | up |
| Cadaverine | 0.12 | 0.07 | 1.582 | 0.017 | -0.744 | down |
| Palmitic Acid | 0.13 | 0.21 | 1.555 | 0.045 | 0.617 | up |
| LPB vs LPC | | | | | | |
| Metabolites | LPB | LPC | VIP | *q*-value | Log_2_FC | Trend |
| Gentisic acid | 0.87 | 1.76 | 1.537 | 0.038 | 1.009 | up |
| 3-Hydroxyphenylacetic acid | 0.24 | 0.91 | 1.590 | 0.039 | 1.945 | up |
| 2-Aminobutyric acid | 0.02 | 0.01 | 1.445 | 0.049 | -0.457 | down |
| 5-Hydroxyindole-3-acetic acid | 0.02 | 0.01 | 1.473 | 0.036 | -0.594 | down |
| L-Arginine | 0.02 | 0.01 | 1.742 | 0.011 | 1.300 | up |

^1^ variable importance in projection;

^2^ fold change;

Treatments: CON is 16.7% CP based on nutritional requirements, CP of LPA, LPB and LPC is decreased by ~10%, SP proportion (% of CP) 21.2, 25.9 and 29.4 respectively.
